# Clinical Lectures on Abdominal Hernia

**Published:** 1893-09

**Authors:** 


					C /?
llcal Lectures on Abdominal Hernia.
By William H.
-Bennett. Pp. ix., 225. London : Longmans,
t^Een, & Co. 1893.
Hos -ls is a collection of clinical lectures given at St. George's
y^ry at irregular intervals, and now brought together in a
e are^able form, and illustrated with twelve diagrams.
Vafiou 0r's suggestions as to the best mode of treating the
PfactiS implications met with in herniotomy are good and
COn a ^0r the most part, although he seems to err on the side
Plan wervatism in dealing with gangrene of the bowel. The
%rn *ch he advocates is to divide the stricture freely, and
* ^r8e h ?anSrenous bowel just inside the abdomen, leaving
jj ^rainage tube in such a way that its inner extremity
h^r m a^rn?st in contact with the reduced bowel. He " has
with a case in which the operation of resection of
^tie^Vas worthy of serious thought, as he always found the
ln too critical a state constitutionally to bear the
C's'?n *7 Pr?l?ngation of the operation entailed by the ex-
ot the affected parts." Two cases are given where
202 REVIEWS OF BOOKS.
s
gangrenous gut was treated with success by the author
method; but the tendency of modern surgery is in ^
direction of resection in all these cases, and with furtj1 ^
simplification of details of operating we have little doubt tn
this will be the plan of the future. The author very proper.J
insists upon the importance of performing the radical cure ^
all cases of herniotomy, and describes very fully his mode
operating. We think that he lays too much stress upon *
mere removal of the hernial sac as a means of radical cn*^
indeed he seems to consider that in children nothing ni?re.
necessary in order to effect a permanent cure of the her111
We should be sorry ourselves to rely upon this alone. ,y
In operating upon adults the author follows pretty
the method described by Mr. Barker,1 the only difference be1 <j
a slight variation in the mode of suturing the divided her11
ai*y
tiofl
sac to the abdominal parietes. No reference is made to
previously described method of operating, with the excep
of the following paragraph (p. 196): 6
" My first experience of the method described was in a ca .
of strangulated inguinal hernia about three and a half y?,fi
ago; but, so far as any published record is concerned, 0
Stanmore Bishop was the first, I believe, to call attention
the radical cure by complete invagination of the sac by ^
open operation in the Lancet of March (sic) 31, 1890, where j
describes a well-considered method, in which the sac is
completely inside out and fixed across the mouth of the ingul .
canal by means of a purse-string-like arrangement of sutu1?
in such a way that the wrinkling up of the sac produces a ' ^
exactly over the internal ring.' " ^
As to which we would remark that in the article thus alju *
? to, published in May and not in March, Mr. Stanmore J
gives all credit for the introduction of the invagination fie
to Dr. Macewen, who had given a full account of it in
Annals of Surgery and in the British Medical Journal for 1887- 0[
It does not much matter who was the first to perform an^ut
the numerous operations for the radical cure of hernia! \\
credit ought certainly to be given to Macewen and to
Banks for being pioneers in demonstrating the safety
certainty of operating by the open method. $
The lectures will well repay perusal, being full of val^le
and practical suggestions by one who has had consider
experience.
1 Brit. Med. Journ., Dec., 1887.

				

